# Role of Bile Acids in the Regulation of Food Intake, and Their Dysregulation in Metabolic Disease

**DOI:** 10.3390/nu13041104

**Published:** 2021-03-28

**Authors:** Cong Xie, Weikun Huang, Richard L. Young, Karen L. Jones, Michael Horowitz, Christopher K. Rayner, Tongzhi Wu

**Affiliations:** 1 Adelaide Medical School, Center of Research Excellence (CRE) in Translating Nutritional Science to Good Health, The University of Adelaide, Adelaide 5005, Australia; c.xie@adelaide.edu.au (C.X.); weikun.huang@adelaide.edu.au (W.H.); richard.young@adelaide.edu.au (R.L.Y.); karen.jones@adelaide.edu.au (K.L.J.); michael.horowitz@adelaide.edu.au (M.H.); chris.rayner@adelaide.edu.au (C.K.R.); 2 The ARC Center of Excellence for Nanoscale BioPhotonics, Institute for Photonics and Advanced Sensing, School of Physical Sciences, The University of Adelaide, Adelaide 5005, Australia; 3 Nutrition, Diabetes & Gut Health, Lifelong Health Theme South Australian Health & Medical Research Institute, Adelaide 5005, Australia; 4 Endocrine and Metabolic Unit, Royal Adelaide Hospital, Adelaide 5005, Australia; 5 Department of Gastroenterology and Hepatology, Royal Adelaide Hospital, Adelaide 5005, Australia; 6 Institute of Diabetes, School of Medicine, Southeast University, Nanjing 210009, China

**Keywords:** bile acids, TGR-5, FXR, gastrointestinal hormones, energy intake, body weight, obesity, type 2 diabetes

## Abstract

Bile acids are cholesterol-derived metabolites with a well-established role in the digestion and absorption of dietary fat. More recently, the discovery of bile acids as natural ligands for the nuclear farnesoid X receptor (FXR) and membrane Takeda G-protein-coupled receptor 5 (TGR5), and the recognition of the effects of FXR and TGR5 signaling have led to a paradigm shift in knowledge regarding bile acid physiology and metabolic health. Bile acids are now recognized as signaling molecules that orchestrate blood glucose, lipid and energy metabolism. Changes in FXR and/or TGR5 signaling modulates the secretion of gastrointestinal hormones including glucagon-like peptide-1 (GLP-1) and peptide YY (PYY), hepatic gluconeogenesis, glycogen synthesis, energy expenditure, and the composition of the gut microbiome. These effects may contribute to the metabolic benefits of bile acid sequestrants, metformin, and bariatric surgery. This review focuses on the role of bile acids in energy intake and body weight, particularly their effects on gastrointestinal hormone secretion, the changes in obesity and T2D, and their potential relevance to the management of metabolic disorders.

## 1. Introduction

**Figure 1:**
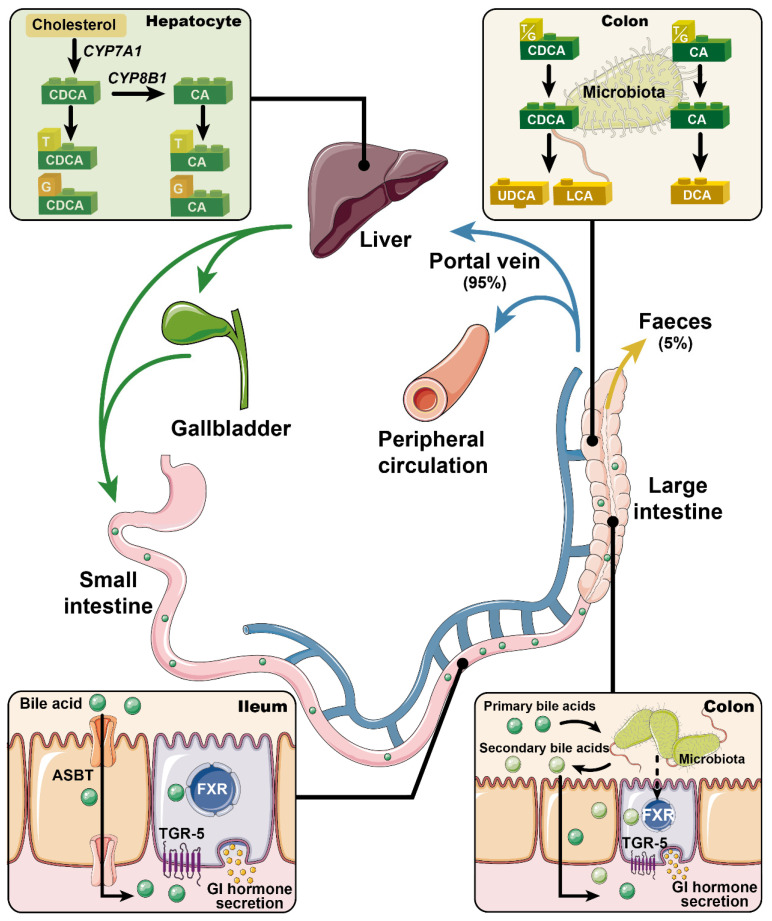
Primary bile acids (i.e., chenodeoxycholic acid (CDCA) and cholic acid (CA)) are synthesized from cholesterol in the liver, and conjugated to glycine and taurine prior to their secretion into bile. In response to meals, bile acids are discharged into the intestine. Approximately 95% of the intestinal bile acids are absorbed in the ileum via apical sodium bile acid co-transporter (ASBT) and return to the liver for re-secretion (i.e., the enterohepatic circulation). Only ~5% of bile acids escape into the large intestine and are modified by gut microbiota into secondary bile acids (e.g., deoxycholic acid (DCA), lithocholic acid (LCA), and ursodeoxycholic acid (UDCA)). Bile acids are now recognized as pivotal signaling molecules that participate in the regulation of metabolic homeostasis through regulating the secretion of gastrointestinal hormones. This complex process has been linked to activation of the nuclear farnesoid X receptor (FXR) and/or the membrane Takeda G-protein-coupled receptor 5 (TGR5). Accordingly, modulation of FXR and/or TGR5 signaling has been actively pursued for the management of metabolic disorders.

Bile acids are synthesized in the liver, where cholesterol is converted via 7α-hydroxylase (CYP7A1) and, to a lesser extent, 27α-hydroxylase (CYP27A1) and 24-hydroxylase (CYP46A1), to the primary bile acids cholic acid (CA) and chenodeoxycholic acid (CDCA) in humans (CA and muricholic acid in rodents). These are then conjugated to glycine or taurine, prior to their secretion into bile [1]. Following meal ingestion, bile acids are released into the gut upon gallbladder emptying, and about 95% of intestinal bile acids is absorbed in the ileum via the apical sodium bile acid co-transporter (ASBT), returning to the liver for re-secretion—a highly efficient process known as “enterohepatic circulation”. A small fraction of bile acids reach the large intestine, where they are modified (through de-conjugation and dihydroxylation) by gut bacteria to secondary bile acids such as deoxycholic acid (DCA), lithocholic acid (LCA), and ursodeoxycholic acid (UDCA, a secondary bile acid in humans, but a primary bile acid in rodents), and absorbed passively into the circulation or excreted in the feces [2] (Figure 1). Bile acids lost to the large intestine are replenished by de novo hepatic synthesis, which is regulated by fibroblast growth factor-19 (FGF19) signaling in the small intestine in humans (or FGF15 in rodents). Thus, bile acids are found in high concentrations in the liver [3], bile [4], and small intestine [5].

For more than a century, bile acids have been regarded solely as “intestinal detergents” that emulsify dietary fat for digestion and transport. The recognition that bile acids are also pivotal signaling molecules orchestrating glucose, lipid and energy metabolism is recent. Bile acids also bind to numerous nuclear and cytoplasmic receptors such as the vitamin D receptor [6], pregnane X receptor [7], and constitutive androstane receptor [8]. However, it was the identification of the bile acid-specific nuclear farnesoid X receptor (FXR) in 1999 and membrane Takeda G-protein-coupled receptor 5 (TGR5) in 2002 that provided a mechanistic framework for a role of BA signaling in the context of metabolism [9,10]. FXR and TGR5 are present in numerous tissues including the central and peripheral nervous systems; bile acid signaling in the latter has been shown to regulate energy intake [11], as supported by the observation that suppression of energy intake induced by intravenous injection of DCA is attenuated when TGR5 was silenced in the vagal nodose ganglia in rats [12]. However, the clinical relevance of this concept is unclear, particularly given that plasma bile acid concentrations are low and that in obese individuals, relative elevation in plasma bile acid levels are not associated with reduced energy intake. In line with the high turnover of bile acids in the enterohepatic circulation, both FXR and TGR5 are expressed abundantly in the liver and the intestine. Signaling through both receptors has been linked to the secretion of gastrointestinal hormones, known to be integral to the maintenance of metabolic homeostasis (Figure 1). For example, the release of ghrelin from gastric G-cells during fasting appears pivotal to sensations of hunger, and stimulation of energy intake. After meals, the secretion of cholecystokinin (CCK) from enteroendocrine I-cells located in the upper gut, and glucagon-like peptide-1 (GLP-1) and peptide YY (PYY) from L-cells located most abundantly in the distal gut, form an integrated signaling system that slows gastrointestinal motility and transit, drives the secretion of insulin to regulate postprandial glucose metabolism (via GLP-1) and suppresses appetite and energy intake [13]. The role of bile acids in the control of blood glucose and lipid metabolism has been reviewed in detail [14,15,16,17], but their potential to impact on the regulation of energy intake has received less attention, despite the recognition, since 1968, that oral administration of CDCA and DCA stimulated PYY secretion and suppressed appetite in obese individuals [18]. The current review addresses the effects of bile acids on gastrointestinal hormone secretion, energy intake, and body weight as well as the relevance of bile acid dysregulation in obesity and type 2 diabetes (T2D).

## 2. Effects of Bile Acids on Gastrointestinal Hormone Secretion

The last two decades have witnessed a substantial effort to increase the understanding of the effects of bile acids on gastrointestinal hormone secretion and the consequent impact on metabolism. In healthy individuals, postprandial plasma bile acid concentrations have been reported to correlate negatively with ghrelin, and positively with GLP-1 and PYY [19]. Similar relationships have also been observed in obese patients following bariatric surgery [20]. However, bile acids per se do not appear to affect ghrelin secretion in rats; intestinal infusion of a mixture of physiological bile acids did not affect portal ghrelin levels [21]. In contrast, small intestinal sensing of bile acids has been reported to inhibit CCK secretion in both rodents and humans [22,23], supporting the existence of a negative feedback loop between the two. In contrast, the effects on GLP-1 and PYY release from L-cells have been studied extensively in preclinical and clinical models [24,25,26], stimulating the potential development of bile acid-based interventions for metabolic disorders. While bile acid-induced release of GLP-1 and PYY has been linked to signaling via FXR and TGR5, the data are inconsistent, which may relate to differences in the binding affinity of individual bile acids at FXR and TGR5 (Table 1) and/or complex interactions between the two signaling pathways.

### 2.1. FXR

FXR is expressed abundantly in the liver and the intestine, and the binding affinity of individual bile acids is variable (CDCA > DCA > LCA > CA > UDCA, Table 1). FXR was initially identified as a regulator of bile acid metabolism [14], and subsequently as a modulator of L-cell secretion. Indeed, FXR is expressed by the murine L-cell line, GLUTag. However, the FXR agonist GW4064 and CDCA (which preferentially binds FXR) were shown to suppress glucose-induced proglucagon expression and GLP-1 secretion in this cell line by decreasing glycolysis, whereas silencing FXR abolished these effects [27]. These observations have been replicated in studies with different L-cell lines (i.e., NCI-H736 [28] and STC-1 [29]). In a similar manner, GW4064 blunted the GLP-1 response to short-chain fatty acids (SCFA) in both GLUTag and NCI-H716 cell lines [30]. Consistent with these observations, FXR-deficient mice exhibited increased GLP-1 secretion in response to both dietary fiber, which increases colonic SCFA [30], and oral glucose [31]. Oral intake of GW4064 (10 mg/kg, 2 doses over 12 h) also decreased active GLP-1 levels in the plasma of mini-pigs [28]. However, in an isolated perfusion model of rat intestine, both luminal and vascular perfusion of GW4064 failed to affect the GLP-1 response to a physiological mixture of bile acids in rats [21]. In mice, diversion of bile acids from the gallbladder to the ileum was shown to modestly increase GLP-1 secretion, improve glucose tolerance, and induce weight loss [32]. The reductions in postprandial blood glucose and body weight induced by this procedure were abolished in intestinal FXR-knockout mice, suggesting that intestinal FXR-signaling can potentially promote GLP-1 secretion. Unfortunately, the study failed to determine whether the rise in GLP-1 was specifically induced by FXR-activation [32]. Of note, oral administration of the intestine-restricted FXR agonist, fexaramine, in mice was reported to increase the abundance of LCA-producing gut bacteria to activate TGR5-signaling indirectly, leading to enhanced GLP-1 secretion and improvement in insulin sensitivity and lipid profile as well as the promotion of adipose tissue browning [33]. Accordingly, outcomes derived from ex vivo and in vivo experiments are, by and large, inconsistent, although the intestine-restricted FXR signaling appears to have an overall favorable effect on metabolic health.

### 2.2. TGR5

TGR5, also known as GPBAR1, is a G-protein coupled receptor that is expressed widely in the gastrointestinal tract, pancreas, liver, gallbladder, and adipose tissue. Like FXR, its binding affinity for individual bile acids varies substantially (LCA > DCA > CDCA > CA > UDCA, Table 1) [34]. TGR5 activation has been reported to suppress hepatic macrophages, induce gallbladder relaxation and refilling, and promote intestinal motility [14]. TGR5 is also expressed on L-cells. Unlike FXR, stimulation of TGR5 by LCA and DCA was shown to potently stimulate GLP-1 secretion from STC-1 cells in a dose-dependent manner, an effect suppressed by downregulation of TGR5 expression [35]. The stimulatory effect of TGR5 on GLP-1 secretion required the closure of ATP-sensitive potassium (KATP) channels and elevated intracellular concentrations of cAMP and Ca2+ [36,37]. A major observation in relation to TGR5 signaling was the demonstration of its basolateral location on L-cells. Thus, to activate TGR5, it is necessary for bile acids or other TGR5 ligands to be transported through the epithelial layer [38]. However, the readily absorbed TGR5 agonist SB-756050 failed to stimulate GLP-1 secretion significantly, or improve glycemic control at various doses compared with the placebo in acute studies involving patients with T2D [39]. It is noteworthy that L-cells are distributed most densely in the distal gut regions [13]. It would therefore be of interest to investigate whether delivery of TGR5 agonists should be targeted at the distal gut.

PYY is co-released with GLP-1 from L-cells, and it was initially noted that perfusion of DCA (1–25 mM) into the isolated rabbit colon increased PYY secretion substantially in a dose-dependent manner [18]. Intracolonic administration of DCA or TCA in humans has also been shown to induce a rapid and substantial rise in plasma PYY [40,41,42]. Similar to TGR5-mediated GLP-1 secretion, the outcomes of studies using isolated rat colon indicate that bile acid-induced PYY secretion is dependent on bile acid translocation from the luminal to basolateral side [43]. That PYY secretion is less evident in response to bile acids with poor affinity to TGR5, and attenuated in TGR5-knockout models, attests to the fundamental relevance of TGR5-signaling to bile acid-induced PYY secretion [44].

In summary, there is compelling evidence for a role of bile acids in the modulation of GLP-1 and PYY secretion in both animals and humans. Stimulation of TGR5 on L-cells induces the secretion of both hormones, while effects of FXR signaling remain controversial. The interactions between FXR and TGR5 signaling remain poorly characterized and an improved understanding may be of relevance to the development of novel strategies for the management of metabolic disorders.

## 3. Effects of Bile Acid Signaling on Energy Intake and Body Weight

In light of the effects of bile acids on appetite regulation, particularly via the secretion of gastrointestinal hormones, it is intuitively likely that modulating bile acid signaling affects energy balance. Genetic ablation of the bile acid synthesis enzyme CYP8B1, leading to a deficiency of 12α-hydroxylated bile acids (e.g., CA), has been shown to be associated with reduced energy intake and subsequent weight gain in mice fed a fat enriched diet [48,49]. However, these effects appeared to be secondary to impaired fat hydrolysis and the increased exposure of unabsorbed fat to the distal gut, as in these mice, there was an increase in energy intake when fed a fat-free diet [49]. Nevertheless, this study supports the fundamental role of endogenous bile acids in fat digestion and absorption, which may influence energy intake and body weight indirectly.

**Figure 2:**
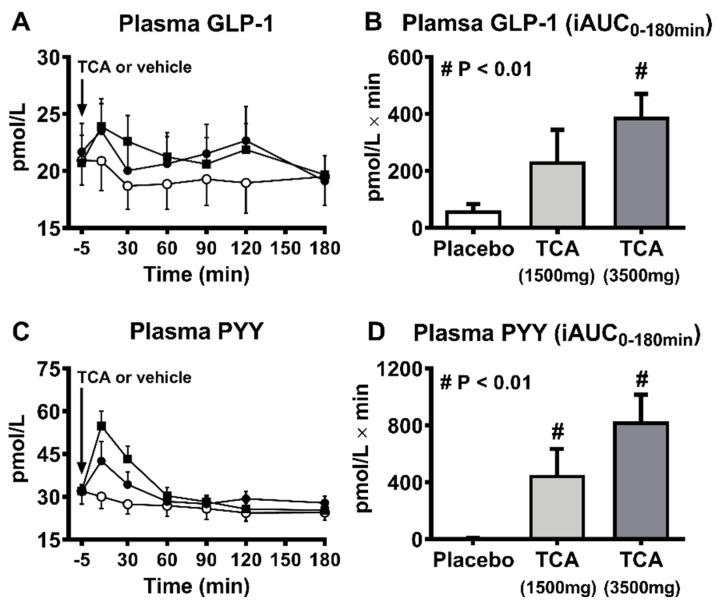
Plasma glucagon-like peptide-1 (GLP-1) (**A**,**B**), and peptide YY (PYY) (**C**,**D**) (means ± sem.) after rectal taurocholic acid (TCA) enema in 10 healthy humans. (**B**) *p* = 0.019 for incremental area under the curves (iAUC); r = 0.48, *p* = 0.004 for dose-dependent effect; (**D**) *p* = 0.0005 for iAUC; r = 0.56, *p* = 0.001 for dose-dependent effect. Reproduced with permission from [42] © (2013).

The outcomes of preclinical and clinical studies involving administration of various bile acids have been equivocal in relation to effects on energy intake and body weight (Table 2). For example, supplementation with CA or UDCA prevented weight gain in mice fed a high-fat diet [50,51,52], possibly reflecting a TGR5-related increase in energy expenditure [50]. Moreover, a number of other bile acid species with high affinity for TGR5 including hyocholic acid (HCA), hyodeoxycholic acid (HDCA), DCA, and TCA failed to affect energy intake or body weight in rodents with or without diabetes [28,53,54]. Information relating to the effects of bile acids on appetite and energy intake in humans are limited. In healthy individuals, rectal administration of TCA substantially stimulated GLP-1 and PYY secretion and suppressed hunger in a dose-dependent manner [42]. Similarly, in obese individuals with T2D, rectally administered TCA significantly suppressed energy intake dose-dependently [41]. However, these observations could be confounded by the concurrent urge for defecation induced by rectal TCA perfusion (Figure 2) [42]. More recently, a double-blind, randomized, placebo-controlled 4-week trial that delivered a mixture of encapsulated bile acids (1000mg/day) designed for release in the ileum and colon (to provide dual agonism of FXR and TGR5) showed little effect on body weight in patients with T2D, despite increases in plasma GLP-1 and serum and intestinal bile acids [55].

As discussed, physiological bile acids often activate both FXR and TGR5, but with preferential affinity depending on their molecular structure. Selective FXR- and TGR5-knockout mice, or specific FXR and TGR agonists, have been pivotal to delineation of the respective signaling pathways to the metabolic effects of bile acids. However, outcomes remain inconclusive. Administration of the intestinal FXR agonist, fexaramine, for five weeks to mice fed a high-fat-diet was reported to prevent weight gain. However, this may have reflected an increase in metabolic rate, rather than a reduction in energy intake [56]. In contrast, GW4064 had no effect on either energy intake or body weight in diabetic or obese mice [50,57]. Notably, mice with FXR deficiency (either whole body or intestine-specific knockout) fed a high-fat diet also exhibited reductions in energy intake and body weight compared with wild-type mice [31,58]. Similarly, TGR5 agonism (e.g., by INT-777) was associated with reduced weight gain, apparently by augmenting energy expenditure, without affecting energy intake [36], whereas knockout of TGR5 had no significant effect on body weight or energy intake in mice fed a high-fat diet [36,59]. Clinical outcomes relating to TGR5 or FXR agonism have been disappointing. As discussed, the TGR5 agonist, SB-756050, failed to stimulate GLP-1 secretion or improve glycemic control in individuals with T2D [39]. The effects of TGR5 agonists on energy intake and body weight in humans have not been reported. Treatment with the semi-synthetic FXR agonist, obeticholic acid, over 72 weeks only achieved a modest reduction in body weight (~2 kg) in patients with non-alcoholic fatty liver disease (NAFLD), with or without, T2D [60]. In another 24-week double-blind, randomized, placebo-controlled trial, the non-bile acid FXR agonist, cilofexor, had no effect on body weight in patients with non-alcoholic steatohepatitis [61]. Accordingly, the concept of supplementing bile acids or targeting BA signaling pathways to reduce energy intake and body weight is currently not supported by current clinical evidence.

## 4. Bile Acid Dysregulation in Obesity and T2D

The emerging link between bile acid signaling and the regulation of metabolic homeostasis has stimulated substantial interest in potential phenotypical changes in bile acid profiles in metabolic disorders, particularly obesity and T2D. Although bile acids are present at high concentrations in the liver, bile, and small intestine, bile acid profiles have hitherto been compared in peripheral blood and fecal samples predominantly due to their easy accessibility. Accordingly, processes in relation to small intestinal bile acid transport and absorption are poorly characterized, although gallbladder emptying can be readily assessed using ultrasound.

There is a substantial variation in circulating bile acid levels both between and within individuals [62]. In the context of obesity, most studies have reported that fasting serum/plasma bile acid levels are increased as a result of augmented bile acid synthesis (reflected by an increase in 7α-hydroxy-4-cholesten-3-one (C4)) [63,64,65]. There is evidence that the expression of both hepatic Na+-taurocholate co-transporting polypeptide (NTCP) [66] (responsible for the uptake of bile acids from the portal vein to the liver) and intestinal ASBT is lower in obese individuals [67], and intestinal FGF-19 secretion is also decreased [67,68]. It is, therefore, conceivable that the augmented hepatic bile acid secretion observed during fasting represents a compensatory response to deficiencies in the enterohepatic circulation. In support of this concept, the postprandial increase in circulating bile acids is significantly blunted in obesity [66,69,70] and restored after Roux-en-Y gastric bypass [70]. In addition, the production and fecal excretion of secondary bile acids (e.g., DCA) are increased in obese individuals [71,72], which may be secondary, or contribute to, alterations in gut microbiota (“dysbiosis”) [73], leading to impaired energy metabolism in the host [74]. Obesity-related increases in fasting bile acid levels primarily reflect increases in 12α-hydroxylated bile acids (e.g., CA) [64,66], which are more effective in emulsifying dietary fat than non-12α-hydroxylated bile acids [49]. The shift in the bile acid composition in obesity may, therefore, favor improved fat digestion. Although fasting plasma unconjugated primary bile acids (CA and CDCA) and numerous conjugated primary and secondary bile acids (TCA, GCA, GCDCA, TDCA, and GLCA) are related positively with insulin resistance in obesity [75,76], it remains to be determined whether changes in plasma bile acids represent a manifestation, or the drivers, of obesity.

T2D individuals, with or without obesity, exhibit higher fasting bile acid concentrations in the peripheral circulation compared with non-diabetic controls, mainly due to increased unconjugated and glycine-conjugated DCA and UDCA [64,77,78,79,80]. This rise in plasma secondary bile acids may reflect increased bile acid delivery and a relative abundance of bile acid de-conjugating bacteria in the large intestine [81,82]. Interestingly, the expression of ASBT has been reported to be increased in diabetic rats [83], which would favor enhanced ileal bile acid resorption. However, this does not necessarily lead to increased FGF-19 secretion [77,79,80], or suppression of bile acid synthesis in T2D. Hepatic bile acid synthesis, particularly CA, is, in fact, known to be increased in patients with T2D [80]. In a small group of individuals with T2D (*n* = 15), the plasma BA responses to oral glucose or fat-containing mixed nutrients were reported to be modestly elevated [77]. Gallbladder emptying in this group of patients was similar to healthy controls [84]. However, in this study, T2D patients had relatively poor glycemic control (mean HbA1c = 7.5%) and a long duration of diabetes (6–20 years), with the majority receiving medication (e.g., metformin [85]) known to affect bile acid metabolism.

The magnitude of the increase in fasting bile acids in plasma or serum has been shown to correlate positively with fasting and 2 h-postprandial glucose levels and HbA1c in T2D, and with the degree of insulin resistance in individuals, regardless of the presence of diabetes [79,86]. In a recently reported longitudinal study, 23 bile acid species were analyzed to evaluate their baseline association with incident T2D during a median 3-year follow-up in a large cohort of individuals with normal glucose tolerance [87]. Serum fasting unconjugated primary and secondary bile acids (CA, CDCA, and DCA) were reported to be negatively associated with the risk of T2D, while conjugated primary and secondary bile acids (GCA, TCA, GCDCA, TCDCA, and TUDCA) were positively associated. Moreover, the ratios of conjugated to unconjugated bile acids (TCA/CA, GCA/CA, TCDCA/CDCA, and GCDCA/CA) were positively associated with the development of T2D. These observations support the concept that impaired catalysis of conjugated bile acids by the hepatic bile acid-CoA:amino acid N-acyltransferase (BAAT) [88] and/or intestinal resorption of unconjugated bile acids contribute to the development of T2D. The relevance of postprandial bile acid levels, particularly in the small intestine and liver, to the risk of T2D, however, remains unknown. Further studies are, therefore, required to clarify how bile acid metabolism changes with the progression of glucose dysregulation.

## 5. Relevance of Bile Acids to Therapies for Metabolic Disorders

As discussed, it remains to be clarified whether alterations in bile acids underpin the pathogenesis, or represent a consequence of metabolic derangement. However, there is increasing persuasive evidence to support a role for bile acids in mediating the metabolic benefits of therapies used to treat metabolic disorders including bile acid sequestrants, ASBT inhibitors, metformin, and bariatric surgery.

### 5.1. Bile Acid Sequestrants

Bile acid sequestrants are resins that bind to intestinal bile acids to disrupt their enterohepatic circulation and increase hepatic bile acid synthesis from cholesterol to reduce intestinal secretion of FGF19 (or FGF15 in rodents) [89,90], elevate plasma C4 levels [91], and augment expression of hepatic CYP7A1 [89,91,92]. The increase in de novo bile acid synthesis is sufficient to maintain the size of the total bile acid, but often changes its composition [80,93]. For example, in T2D patients, treatment with colesevelam (3.75 g/day) over eight weeks increased CA, but decreased CDCA and DCA [80], shifting the bile acid pool toward a more hydrophilic phenotype. Due to their effects on the enterohepatic circulation, bile acid sequestrants were initially developed to treat hypercholesterolemia. Surprisingly, they were also shown to be associated with a substantial improvement in glycemic control in patients with T2D, leading to potential re-purposing for the management of T2D [94], although the mechanism of their glucose-lowering action remains elusive. Several preclinical and clinical studies have reported a significant increase in GLP-1 secretion, associated with the use of bile acid sequestrants [89,93,95], although some studies have reported minimal [96,97], or the opposite effect [97,98]. Similarly, evidence for the effects of bile acid sequestrants on energy intake and energy expenditure is also inconsistent. In high-fat fed C57BL/6J mice, the bile acid sequestrant, colestimide, was reported to increase energy expenditure in brown adipose tissue and prevent diet-induced obesity, without affecting energy intake or lipid absorption [90]. In a similar study of hyperlipidemic transgenic mice, colestilan was reported to reduce body weight, accompanied by an increase in energy intake, a reduction in total energy expenditure, and enhanced carbohydrate catabolism [99]. In clinical trials of healthy individuals and patients with obesity and/or T2D, bile acid sequestrants have been found to be weight-neutral [93,100,101,102]. While further studies are required to clarify the glucose-lowering mechanisms of bile acid sequestrants, the latter do not appear to be an effective treatment for obesity.

### 5.2. Apical Sodium Bile Acid Co-Transporter (ASBT) Inhibitors

Similar to bile acid sequestrants, ASBT inhibitors impair intestinal bile acid resorption, leading to increased delivery of bile acids to the large intestine and decreased bile acid concentrations in the circulation [103,104,105,106]. These agents were first developed to treat hypercholesterolemia, but were subsequently applied to the management of functional constipation and non-alcoholic steatohepatitis [107]. While inhibition of ASBT remarkedly increases GLP-1 secretion in both rodents [108] and humans [109], ASBT inhibitors have not affected the energy intake or body weight in animals [104,106]. Their effect on energy intake in humans has not been reported.

### 5.3. Metformin

Metformin remains the first-line therapy for glucose-lowering in T2D [85], but also suppresses appetite and reduces body weight modestly [110,111,112,113]. The potential for metformin to increase plasma GLP-1 and PYY levels has been widely recognized in both preclinical and clinical studies [114,115,116,117]. There is evidence that the latter may be attributable, at least in part, to the inhibition of intestinal bile acid resorption by metformin. Indeed, metformin substantially decreases serum FGF-19, and increases fecal bile acid excretion and serum C4 levels in T2D [118]. In high-fat-fed mice, metformin was also shown to prevent weight gain, apparently by increasing energy expenditure through upregulation of the thermogenic gene (Ucp1) in white adipose tissue, without affecting energy intake [118]. That the effect on body weight was abolished in mice with intestinal-specific FXR knockout supports an important role for intestinal FXR signaling in metformin-induced weight loss in mice [118]. Moreover, metformin modifies the gut microbiota [119]; metformin therapy (1700 mg/day) over four months results in major shifts in over 50 bacterial strains, which may account for glucose-lowering in T2D [113]. In mice, weight loss induced by metformin may be attributable to a reduction in intestinal Bacteroides fragilis and resultant increases in GUDCA; the latter antagonizes FXR signaling to improve glucose metabolism and reduce body weight [118]. In this context, delayed-released metformin (of minimal intestinal absorption) may be desirable to maximize the interaction between metformin and the gut microbiota for the management of T2D.

### 5.4. Bariatric Surgery

Despite emerging pharmaceutical treatments, bariatric surgery remains the most effective intervention for obesity and T2D. Relative to adjustable gastric banding and sleeve gastrectomy, procedures that bypass segments of the small intestine (e.g., Roux-en-Y gastric bypass, duodenal-jejunal bypass, and biliopancreatic diversion) are in general more effective [120,121]. While the underlying mechanisms remain incompletely understood, emerging evidence suggests that the expedited flow of bile acids to the distal gut may be important. Indeed, bile acid diversion from the duodenum to distal ileum increases GLP-1 [122], decreases blood glucose [32,122,123], and reduces body weight substantially [32,123] in rodents with diet-induced obesity. While the expression of bile acid receptors (i.e., TGR5 and FXR) in the distal gut is not affected by bariatric surgery [123], increased delivery of bile acids into the large intestine may alter the composition of the gut microbiome after bariatric surgery (or vice versa) [32,123,124], thereby influencing host energy metabolism [125]. That FXR-knockout abolishes [32,58], while TGR5 knock-out preserves [32], the weight loss effect of Roux-en-Y gastric bypass or ileal biliary diversion in high-fat-fed mice, suggests that FXR, but not TGR5, signaling is indispensable for weight loss induced by the diversion of bile acids to the distal small intestine. However, the significance of FXR signaling in humans is questionable, since the administration of the FXR agonist, obeticholic acid, over 72 weeks, showed little effect on body weight in patients with NAFLD [60].

## 6. Concluding Comments

The recognition of bile acids as important signaling molecules that orchestrate metabolic homeostasis through specialized receptors (FXR and TGR5) has stimulated active research to determine their relevance to the pathogenesis of, and therapeutic potential for the management of, metabolic disorders. Recent studies, focusing on the enterohepatic circulation and bile acid sensing, are indicative of major shifts in plasma and fecal bile acid profiles in obesity and T2D, and of the potent effects of bile acids on GLP-1 and PYY secretion from enteroendocrine L-cells. Accordingly, assessment of the bile acid profile may be of relevance to predict the risk of obesity and T2D, while targeting bile acid signaling pathways may represent an attractive strategy for the prevention and management of these metabolic disorders. The efficacy of bile acids to stimulate gut hormone secretion is related to their affinity for TGR5 and FXR; activation of TGR5 (expressed on the basolateral side of the L-cells) mediates bile acid-induced GLP-1 and PYY secretion, whereas FXR signaling has been shown to suppress these actions, or modify TGR5 signaling indirectly, while studies of physiological bile acids or agonists of TGR5 and FXR have yielded inconsistent outcomes on blood glucose, energy intake, and body weight changes in both animal and human studies. However, several interventions with proven benefits on metabolic health are clearly associated with disrupted, or potentially accelerated, enterohepatic circulation. Studies are now warranted to determine whether there are causal links between the bile acid profile and metabolic outcomes and, if so, the underlying mechanisms. Finally, it would also be of interest to explore whether bile acids have additive or synergistic effects with other (dietary or pharmaceutical) interventions to promote weight loss and glycemic control.

## Figures and Tables

**Table 1:** Binding affinities of bile acids to human TGR5 and FXR.

Bile Acid	TGR5			FXR		
	Subjects	Indicator	EC_50_	Subjects	Indicator	EC_50_
**Primary Bile Acids**						
**CA**	CHO cells/HEK293	Intracellular cAMP	7.72 µM [34]/ >10 µM [10]	CV-1 cells	Reporter gene activation	No effect [45]
**CDCA**	CHO cells/HEK293	Intracellular cAMP	4.43 µM [34]/ 4 µM [10]	HepG2 cells /CV-1 cells	Reporter gene activation	10 µM [9]/ 50 µM [45]
	CHO cells	Reporter gene activation	6.71 µM [46]	Cell-free	Ligand-sensing assay	4.5 µM [47]
**Conjugated Primary Bile Acids**						
**TCA/GCA**	CHO cells	Reporter gene activation	4.95 µM/ 13.6 µM [46]	Cell-free	Ligand-sensing assay	No effect [47]
**TCDCA/** **GCDCA**	CHO cells	Reporter gene activation	1.92 µM/ 3.88 µM [46]	Cell-free	Ligand-sensing assay	10 µM [47]
**HCA**				Cell-free	TR-FRET FXR coactivator assay	70.06 µM (IC_50_) [28]
**Secondary bile acids**						
**DCA**	CHO cells	Intracellular cAMP	1.01 µM [34]	HepG2 cells	Reporter gene activation	100 µM [9]
	HEK293	Intracellular cAMP	575 nM [10]	CV-1 cells	Reporter gene activation	50 µM [45]
**LCA**	CHO cells	Intracellular cAMP	0.53 µM [34]	CV-1 cells	Reporter gene activation	50 µM [45]
	HEK293	Intracellular cAMP	35 nM [10]	Cell-free	Ligand-sensing assay	25 µM [6]
**UDCA**	CHO cells	Reporter gene activation/Intracellular cAMP	36.4 µM [46]/ No effect [34]	CV-1 cells	Reporter gene activation	No effect [45]
**HDCA**	CHO cells	Reporter gene activation	31.6 µM [46]	Cell-free	TR-FRET FXR coactivator assay	62.43 µM [28] (IC_50_)
**Conjugated Secondary Bile Acids**						
**TDCA/** **GDCA**	CHO cells	Reporter gene activation	0.79 µM /1.18 µM [46]	Cell-free	Ligand-sensing assay	500 µM [47] (IC_50_)
**TLCA/** **GLCA**	CHO cells	Reporter gene activation	0.29 µM /0.54 µM [46]	Cell-free	Ligand-sensing assay	3.8 µM/4.7 µM [47] (IC_50_)
**TUDCA/** **GUDCA**	CHO cells	Reporter gene activation	30.0 µM /33.9 µM [46]	Cell-free	Ligand-sensing assay	No effect [47]
**THDCA/GHDCA**	CHO cells	Reporter gene activation	24.2 µM/36.7 µM [46]			

Note: EC50: the concentration for a half maximal effect; IC50: the concentration for a half maximal inhibitory effect; CHO: Chinese hamster ovary cells; HepG2 cells: Human hepatoma cell line; CV-1 cells: Monkey kidney fibroblast cells (CV-1 line); HEK293: human embryonic kidney cell line 293; TR-FRET FXR coactivator assay: commercial assay kit for screening ligand for FXR.

**Table 2:** Reported effects of bile acids on energy intake and body weight in preclinical and clinical models.

Bile Acid		Model	Dose	Method	Effect	Ref
**Conjugated Bile Acid**						
**Primary**	**TCA**	HFD Sprague-Dawley rat + streptozotocin	0.05% or 0.3%	Fed with high-fat diet for 12 weeks	Body weight − Energy intake −	[54]
		Patients with T2DM	0.66, 2, 6.66, or 20 mmol	Rectal administration	Energy intake ↓ (~47% at 20 mmol)	[41]
	**HCA**	db/db mice; HFD C57BL/6J mice + streptozotocin; C57BL/6J mice	100 mg/kg/day	Oral gavage for 28 days	Body weight − Energy intake −	[28]
	**TUDCA**	db/db mice; HFD C57BL/6J mice + streptozotocin; C57BL/6J mice	100 mg/kg/day	Oral gavage for 28 days	Body weight − Energy intake −	[28]
**Secondary**	**HDCA**	db/db mice; HFD C57BL/6J mice + streptozotocin; C57BL/6J mice	100 mg/kg/day	Oral gavage for 28 days	Body weight − Energy intake −	[28]
**Unconjugated Bile Acid**						
**Primary**	**CA**	C57BL/6J mice	0.5%	High-fat diet fed for 47 days	Body weight ↓ (24%) Energy expenditure ↑ (~50%) Energy intake −	[50]
			0.5%	High-fat diet fed for 9 weeks	Body weight ↓ (6g, ~18%) Energy expenditure ↑ (29%) Energy intake ↑ (20%)	[52]
	**UDCA**		0.5%	High-fat diet fed for 8 weeks	Body weight ↓ (15%)	[51]
**Secondary**	**DCA**	C57BL/6J mice	0.1%	High-fat diet fed for 3 weeks	Body weight − Energy intake −	[53]

Note: EC50: the concentration for a half maximal effect; IC50: the concentration for a half maximal inhibitory effect; CHO: Chinese hamster ovary cells; HepG2 cells: Human hepatoma cell line; CV-1 cells: Monkey kidney fibroblast cells (CV-1 line); HEK293: human embryonic kidney cell line 293; TR-FRET FXR coactivator assay: commercial assay kit for screening ligand for FXR. Cholic acid (CA); Chenodeoxycholic acid (CDCA); Taurocholic acid (TCA); Glycocholic acid (GCA); Taurochenodeoxycholic acid (TCDCA); Glycochenodeoxycholic acid (GCDCA); Hyocholic acid (HCA); Deoxycholic acid (DCA); Lithocholic acid (LCA); Ursodeoxycholic acid (UDCA); Hyodeoxycholic acid (HDCA); Taurodeoxycholic acid (TDCA); Glycodeoxycholic acid (GDCA); Taurolithocholic acid (TLCA); Glycolithocholic acid (GLCA); Tauroursodeoxycholic acid (TUDCA); Glycoursodeoxycholic acid (GUDCA); Taurohyodeoxycholic acid (THDCA); Glycohyodeoxycholic acid (GHDCA).
